# Home gardening and fruit and vegetable intake in rural settlements in Northeast Hungary

**DOI:** 10.1038/s41598-026-39593-2

**Published:** 2026-02-09

**Authors:** Anita Simon, Helga Bárdos

**Affiliations:** 1 Doctoral School of Health Sciences, University of Debrecen, Debrecen, Hungary; 2 Department of Public Health and Epidemiology, Faculty of Medicine, University of Debrecen, Kassai u. 26, Debrecen, 4029 Hungary

**Keywords:** Home gardening, Fruit and vegetable intake, Rural areas, Northeast hungary, Epidemiology, Risk factors

## Abstract

Several studies have found that home gardening can impact fruit and vegetable intake. In Hungary, where fruit and vegetable consumption is among the lowest in the European Union (EU), poor diet is the main behavioral risk factor contributing to mortality. Therefore, this study explored the associations between home gardening and fruit and vegetable intake, as well as other health-related factors, in two rural settlements in Northeast Hungary. Participants for the cross-sectional study were recruited from two small rural towns (*n* = 269). The online survey collected demographic data, dietary habits, physical activity, and health status. We used multivariable logistic regression analysis to examine the associations between home gardening and the odds of meeting fruit and vegetable intake recommendations. Almost two-thirds of the respondents grew fruit and vegetables at home. Most gardeners were women, highly educated, married, and had children under the age of 18. One-quarter of participants (24.9%) met the dietary recommendation for daily fruit and vegetable consumption, and 86.6% of them had a home garden. Participants with a home garden were more than four times more likely to meet the guidelines for fruit and vegetable intake than those without one (AOR = 4.49, 95% CI 1.95–10.18). This study provides evidence of a strong positive association between home gardening and meeting fruit and vegetable intake recommendations in rural Northeast Hungary. By focusing on an under-researched population with low baseline consumption, the findings extend prior research and support the potential role of home gardening as a context-specific public health strategy to improve dietary behaviors.

## Background

Diet low in fruits and vegetables is among the leading dietary risk factors that contribute greatly to morbidity and mortality from chronic noncommunicable diseases, including cardiovascular diseases, type 2 diabetes, and cancer^1^. According to the GBD Diet study, in 2017 globally 11 million deaths were attributed to dietary factors, 2 million to low intake of fruits, and 1.5 million to low intake of vegetables^1^. The OECD State of Health in the EU reported in 2021 that approximately half of all deaths in Hungary can be attributed to behavioral risk factors, among which a poor diet accounts for the largest part of mortality, followed by tobacco smoking. In Hungary, one-quarter of all deaths in 2019 could be attributed to dietary risks, which is above the EU average of 17%^2^. According to the European Health Interview Survey, only 8.2% of people in Hungary reported consuming 5 or more portions of fruit and vegetables daily in 2019^3^.

To reduce the risk of chronic diseases, the optimal intake of 15 dietary factors was calculated considering the lowest risk of mortality from each disease defined by meta-analyses. According to this study, the optimal intake for fruits is 250 g (200–300) per day, and the optimal intake for vegetables is 360 g (290–430) per day^1^. The national nutritional survey revealed that the average daily consumption of fruits was 149 g, and the average daily consumption of vegetables was 190 g per person in Hungary in 2019^4^.

Previous studies have demonstrated that home gardening can increase fruit and vegetable consumption, dietary diversity, and micronutrient adequacy^5–9^. Most evidence comes either from low- and middle-income countries, where gardening has been promoted as a means of improving food security and child nutrition, or from high-income countries, where research has primarily focused on community gardens in urban settings. Gardens can enhance household nutrition through increased consumption of vegetables and fruit and dietary diversification^5–9^. Several studies have confirmed that home gardening correlates with increased consumption of fruits and vegetables, food security, physical activity, weight status, malnutrition, micronutrient insufficiency, general and mental well-being, and time spent outdoors^8,10–17^. Home gardening also positively affects mental and social well-being by bringing people together and sharing work and the goods they produce and by reducing stress^18,19,24^.

Home gardening or allotment gardening was very popular in Hungary in the 1960s and 1980s, when household-level food production played an important role in supplementing limited food supply under the socialist system. However, it has declined since the large-scale urbanization and depopulation of rural settlements that began after the end of socialism. Most people living in post-socialist cities now reside in apartment buildings and do not have their own gardens^20^. In order to recognize the value and potential of home gardens, as well as to increase food security and livelihoods, many governmental, non-governmental, and international organizations have launched initiatives in developing countries to support and increase home gardening activities. Households that produce food for their own consumption can be very important, especially for those living in income poverty^21–23^. The Second European Quality of Life Survey (2009) revealed that domestic food production was common in the new member states of the European Union, where 43% of households produced food. It was more common in rural areas and among those with low incomes^24^.

Hungary represents a relevant setting for such research, given the low levels of fruit and vegetable consumption nationally and the limited evidence on how home gardening may influence dietary behaviors. To our knowledge, no previous quantitative study has examined this association in Hungary. Therefore, the aim of this study was to examine the association between home gardening, fruit and vegetable intake, and other health-related factors among adults living in rural settlements in Northeastern Hungary, an under-researched setting characterized by low baseline consumption. By providing empirical evidence from a rural Hungarian population, this study extends existing literature from Central and Eastern Europe and contributes to a better understanding of home gardening as a potential determinant of fruit and vegetable intake.

## Methods

### Study participants

The participants were from two settlements located in a rural area and a suburb of a city, which are characterized by residential areas with detached houses suitable for cultivating a kitchen garden. Participants were recruited using a convenience sampling method. The survey was promoted through local community channels. Invitation letters were distributed via digital tools, municipal communication platforms, and local residents’ social media groups. Recruitment posters were also displayed in local shops, schools, and post offices, and advertisements were placed in local newspapers. The minimum sample size was calculated using the Cochran formula for sample size calculation for cross-sectional surveys, with 5% margin of error and 95% confidence interval level and with the prevalence of 8.2% of 5 or more portions of daily fruit and vegetable intake. Based on these the minimum sample size was determined to be 116^25,26^.

Data was collected using an online questionnaire between December 2021 and May 2022. Participants accessed the online survey via a link distributed through local Facebook community groups, municipal social media web pages and printed leaflets placed in public locations. The eligibility criteria (being aged 18 or over and residing in one of the study settlements) were presented at the start of the questionnaire, and participants could only proceed after confirming that they met these criteria. No financial or material compensation was provided for participation. Participants provided electronic informed consent before accessing the questionnaire. Completing the questionnaire took approximately 10–15 min.

Most of the questionnaire items (sociodemographic data, dietary habits, nutritional knowledge, physical activity, smoking, weight and height, general health, presence of chronic health problem, activity limitation, well-being) were adapted from a validated instrument, the Hungarian version of the European Health Interview Survey (EHIS). Question related to gardening and nutrition statements were formulated by the research team. They were reviewed by public health nutrition experts. The questionnaire was pilot-tested among 20 adults prior to distribution.

One adult, at least 18 years old, from each household completed the questionnaire. The final sample consisted of 269 households, 94 from the rural area of approximately 500 households and 175 from the suburbs of 4500 households. However, it was not possible to calculate the exact response rate because the link to the questionnaire was shared across community and social media channels.

### Variables

#### Demographics

Sociodemographic data included age, sex, employment status, education (higher education corresponds to college or university level), marital status, household composition, perceived financial situation, and settlement location. All sociodemographic variables were collected using closed questions with predefined response options. Age was entered as an open-ended numeric value (years). Sex (male/female), marital status (single/married/divorced/widowed), employment status (employed/student/unemployed/retired), educational level (primary/secondary/higher) and perceived financial situation (very good/good/average/difficult) were all categorized. Household composition and the number of children under 18 were reported numerically. Settlement location was recorded based on the specific questionnaire link used in each settlement and was later coded as a binary variable.

#### Gardening

The presence of a home garden was assessed by asking: ‘Do you (or anyone in your household) grow vegetables and fruit in a garden for only consumption and not selling?’ with response options of ‘yes’ and ‘no’.

#### Fruit and vegetable intake

Fruit and vegetable consumption was measured by asking about frequency and quantity. The frequency options were “more than once a day,” “once a day,” “six times a week,” “five times a week,” “four times a week,” “three times a week,” “two times a week,” “once a week,” and “less than once a week.” The option for quantity was the average number of servings at one time. Serving sizes were explained using text examples without images. For example, ‘one medium apple or one handful of berries equals one fruit serving’, and ‘one medium pepper equals one vegetable serving’. Frequency responses were converted into daily equivalents (e.g., weekly frequency divided by seven) and multiplied by the reported number of servings per occasion to estimate average daily intake. The variable was dichotomized as ‘not meeting recommendations’ (< 5 servings/day) or ‘meeting recommendations’ (≥ 5 servings/day) according to the WHO recommendation^27^. The recommended daily consumption of vegetables was defined as 3 servings, that of fruit was 2 servings, and dichotomous categories were created accordingly^1^.

#### Nutritional status and general health

The participants were asked to report their weight, height, general health, presence of chronic health problems, activity limitation, smoking behavior (current, previous, never), and satisfaction with life (on a 1–10 scale). The height and weight were entered as open-ended numeric values in centimetres and kilograms. Weight status was calculated according to the WHO BMI cut-off points. General health, chronic health problems and general activity limitations were assessed using the predefined response categories of the EHIS instrument. Self-perceived general health is assessed by a question such as ‘How is your health in general?’ Options were very good, good, fair, poor, and very poor. The presence of chronic health problems was asked, ‘Do you have any longstanding illness or health problem? (By longstanding, I mean illnesses or health problems that have lasted, or are expected to last, for 6 months or more.)’ Options were yes or no. General activity limitations due to health problems were asked ‘For at least the past 6 months, to what extent have you been limited because of a health problem in activities people usually do?’ Options were severely limited, limited but not severely limited, and not limited at all. The coding was present or not present^28^. 

#### Physical activity

The European Health Interview Survey-Physical Activity Questionnaire (EHIS-PAQ) was used to determine the level of participants’ work, transport, and leisure time related moderate to vigorous physical activity (MVPA). Data are reported as follows: work-related MVPA, the percentage of individuals mostly physically active when working; transport-related MVPA, average time (min/week) spent walking or cycling; aerobic MVPA, average time (min/week) of leisure time health enhancing (aerobic) MVPA; muscle strengthening PA, the percentage of individuals performing muscle strengthening physical activity at least 2 days per week; and total MVPA, the percentage of individuals being sufficiently physically active, e.g., at least 150 min/week aerobic physical activity or doing moderate to vigorous work-related PA^29^.

#### Nutrition knowledge score

Five simple statements related to current scientific opinions on diet and health were formulated by the research team to measure the participants’ knowledge of healthy nutrition. These items are widely communicated public health messages and intended to capture general awareness of dietary recommendations to prevent chronic non-communicable diseases. Statements were as follows: (1) Adequate fiber consumption (whole grain cereals, vegetables, fruits) reduces the risk of developing cardiovascular diseases; (2) At least 5 servings of vegetables and fruits should be consumed daily; (3) A healthy diet consists of 5 meals a day; (4) High salt intake can cause high blood pressure and heart disease; (5) Consumption of vegetable oils is healthier than animal fats. Agreement with statements was indicated on a 5-point Likert scale (1 = strongly disagree, 5 = strongly agree). The average score reflected the agreement with the true statements, which were used to indicate a “healthy nutrition knowledge” on a 5-point scale, of which 1 indicates lower and 5 indicates higher knowledge of healthy nutrition. Internal consistency of the nutrition knowledge score was assessed using Cronbach’s alpha. Although the five-item composite score was slightly below the conventional 0.70 threshold, the observed Cronbach’s alpha (α = 0.68) is considered adequate for a newly developed, short scale in an exploratory health study.

#### Well-being index

Participants were also asked, ‘Overall, how satisfied are you with your life these days?’ They had to rate how satisfied they were with their lives on a scale from 1 to 10. Larger values indicated higher extent of satisfaction with life.

### Data analysis

The collected data were coded and analyzed with IBM SPSS 25.0. Descriptive characteristics and comparisons by home gardening status were performed. The distribution of the data is presented as counts and percentages for categorical variables and means (SDs) or medians (IQRs) for continuous variables. The normality of the continuous variables was assessed using the Shapiro–Wilk test and the homogeneity of the variances was examined using Levene’s test. Differences by home gardening status were evaluated using independent samples t-tests for normally distributed variables with equal variances; for non-normally distributed variables, Mann–Whitney U tests were used. Chi-square or Fisher’s exact tests were used for categorical variables. Associations between home gardening and meeting with recommendations for fruit and vegetable intake were assessed using multivariable logistic regression analysis. Multicollinearity was evaluated, variance inflation factors indicated no evidence of multicollinearity among explanatory variables of the model. (VIF range between 1.04 and 1.14). The model fit was assessed using the Hosmer–Lemeshow test, which indicated adequate model fit (χ²(8) = 4.78, *p* = 0.78). The reported odds ratios (95% CIs) were adjusted for sex, age, education, marital status, household composition (having children under age 18), and the location of the settlement. Employment status and perceived financial situation were not included in the final regression model because they did not significantly differ between gardeners and non-gardeners. For categorical variables with more than two categories (e.g., marital status), one category was set as the reference group (e.g., married), and the odds ratios for the other categories were estimated in relation to this reference. Missing data were minimal overall among the study variables, with less than 3% of the data missing. The dependent variables (fruit and vegetable consumption) and the main independent variable (home gardening) in the logistic regression analyses did not contain any missing values. Among the other explanatory variables, there were six missing values for marital status. A complete case analysis was performed, and missing data were handled by listwise deletion.

## Results

### Sociodemographic characteristics of the study participants

Table 1 presents the sociodemographic characteristics of the study participants. Data are shown for all participants and by the presence of a home garden. The average age of the participants was 47.6 years, the majority of the respondents were women (70.3%), more than half (54.3%) had high educational levels, and 73.6% were employed. Most of the study participants were married or lived with a partner, and 35.3% had children under the age of 18 years. The perceived financial situation of 27.9% of the participants was reported as good, 59.4% as fair, and 22.7% as bad. A total of 34.9% of the participants lived in rural areas, and 65.1% lived in suburban areas. Almost two-thirds (64.7%) of the participants cultivated home gardens, with a greater proportion of females than males (68.9% vs. 52.5%), and with more children under 18 years of age (72.6% vs. 60.3%.) More participants with higher educational levels than those with lower educational levels had home gardens (70.5% vs. 57.77%). More participants from rural areas than from suburbs had home gardens. (78.7% vs. 57.1%).

**Table 1:** Sociodemographic characteristics of the study participants by home garden status.

	Total		Have home garden		Do not have home garden		*p* value
	*n*	(%)	*n*	*(%)*	*n*	*(%)*	
**Study participants**	269	(100.0)	174	(64.7)	95	(35.3)	-
**Age (mean**,** SD)**	47.6	(14.3)	48.5	(14.3)	46.1	(13.3)	0.184
**Sex**							
Male	80	(29.7)	42	(52.5)	38	(47.5)	**0.007**
Female	189	(70.3)	132	(68.9)	57	(30.2)	
**Education level**							
High	146	(54.3)	103	(70.5)	43	(29.5)	**0.041**
Low	123	(45.7)	71	(57.7)	52	(42.3)	
**Employment status**							
Currently employed	198	(73.6)	120	(69)	78	(39.4)	0.158
Currently not employed	13	(5.2)	11	(84.6)	2	(15.3)	
Homemaker	8	(3.0)	5	(62.5)	3	(37.5)	
Student	7	(2.6)	7	(100)	0	(0.0)	
Retired	41	(15.2)	29	(70.7)	12	(29.3)	
missing	2						
**Marital status**							
Married	151	(56.1)	114	(75.5)	37	(24.5)	**< 0.001**
Single	42	(15.6)	26	(61.9)	16	(38.1)	
Divorced or widowed	32	(11.9)	12	(37.5)	20	(62.5)	
Living with partner	38	(14.1)	22	(57.9)	16	(42.1)	
missing	6						
**Child under 18**							
Yes	95	(35.3)	69	(72.6)	26	(27.4)	**0.044**
No	174	(64.7)	105	(60.3)	69	(39.7)	
**Perceived financial situation**							
Good	75	(27.9)	46	(61.3)	29	(38.7)	0.486
Fair	160	(59.4)	102	(63.7)	58	(36.3)	
Poor	34	(22.7)	26	(76.4)	8	(23.5)	
**Settlement location**							
Rural	94	(34.9)	74	(78.7)	20	(21.3)	**< 0.001**
Suburb	175	(65.1)	100	(57.1)	75	(42.9)	

The values are presented as numbers and percentages or means (SDs). Differences in the distributions of sociodemographic characteristics by home gardening status were statistically analyzed using the Chi-square test or Fisher’s exact test for categorical variables and the independent-samples t-test for continuous variables (age).

### Fruit and vegetable consumption, physical activity, BMI, nutritional status, and general health of the study participants

#### Fruit and vegetable consumption

The median daily fruit and vegetable consumption of the study participants was 2.3 servings/day, the median daily fruit intake was 1 serving, and the median daily vegetable intake was 1 serving. Participants with a home garden consumed significantly more fruits and vegetables than those without a home garden. The median daily fruit intake was 1.4 servings, and vegetable intake was 1.4 servings among gardeners, whereas among nongardeners, the median daily fruit intake was 0.3 servings, and vegetable intake was 0.6 servings. One-quarter of the participants (24.9%) met the 5 servings/day dietary recommendation for the daily intake of fruit and vegetables, 86.6% of whom had home gardens. (Table 2.)

#### Nutrition knowledge score

The overall mean nutritional knowledge score was 3.7 (SD 0.8) among the study participants and did not differ significantly by home garden status.

#### Physical activity

33% of the participants were mostly physically active when working, of whom 66.6% had a home garden. The median (IQR) transport-related MVPAs were 690 (265–1342) min/week, 687 (303–1362) min/week among gardeners, and 742 (264–1309) min/week among nongardeners, with no significant differences. The average leisure time related aerobic MVPA was 160 min/week, and did not differ by home garden status. 41.6% of participants performed muscle strengthening exercise at least twice a week, of whom 68.6% were gardeners. Approximately 70% of participants were physically active, i.e. performing at least 150 min/week aerobic MVPA or doing physically active work. Physical activity levels did not differ between participants by home gardening status.

#### Nutritional status

The mean BMI of the study participants was 26.7, which exceeded the upper limit of the normal range of 25.0. Among home gardeners, the average BMI was higher than that among nongardeners (27.2 vs. 25.7). The combined prevalence of overweight and obesity was 56.5%, of which overweight was 33.5% and obesity was 23.0%. The distribution of nutritional status groups according to home gardening status did not significantly differ.

#### Smoking

The prevalence of current smokers was 26.4% among the study participants, the prevalence of former smokers was 26.4%, and the proportion of never-smokers was 46.8%. There was no significant difference in smoking status between home gardeners and nongardeners.

#### General health

More than half of the study participants (57.6%) reported very good or good health, 33.0% reported fair, and 8.9% reported poor or very poor health. Self-rated general health did not significantly differ according to home gardening status. One-third (33.1%) of the study participants reported having long-standing health problems, and one-quarter (25.3%) of the participants experienced general activity limitations. These variables did not differ by home gardening status. The well-being of the study participants was 7.6 on a scale ranging from 1 to 10, it was higher (7.7) among gardeners than among nongardeners (7.2).

**Table 2:** Fruit and vegetable consumption, physical activity, BMI, nutritional status and general health of the study participants by home garden status.

	Total		Have home garden		Do not have home garden		*p* value
**Fruit and vegetable consumption**							
Servings/day (median, IQR)	2.3	(1.0-4.7)	3.1	(1.9–5.8)	1.1	(0.3–2.9)	**< 0.001**
Meet the recommendation (n, %)	67	(24.9)	58	(86.6)	9	(13.4)	**< 0.001**
**Fruit consumption**							
Servings/day (median, IQR)	1.0	(0.3-2)	1.4	(0.8–2.1)	0.3	(0.0–1.0)	**< 0.001**
Meet the recommendation (n, %)	88	(32.0)	73	(83.0)	15	(17.0)	**< 0.001**
**Vegetable consumption**							
Servings/day (median, IQR)	1.0	(0.3–2.1)	1.4	(0.3–2.9)	0.6	(0.3–1.4)	**< 0.001**
Meet the recommendation (n, %)	51	(18.9)	43	(84.3)	8	(15.7)	**0.001**
**Nutrition knowledge score** (mean, SD)	3.7	(0.8)	3.8	(0.7)	3.8	(0.9)	0.442
**Physical activity**							
Work-related MVPA (%)	90	(33.4)	60	(66.6)	30	(33.3)	0.801
Transport-related MVPA (min/week; median, IQR	690	(265–1342)	687	(303–1362)	742	(264–1309)	0.549
Leisure time aerobic MVPA (min/week; median, IQR)	160	(38–287)	155	(48–322)	165	(20–260)	0.289
Muscle strengthening PA (%, at least 2 times/week)	112	(41.6)	77	(68.8)	35	(31.3)	0.239
Physically active (%)	188	(69.9)	122	(70.1)	66	(29.1)	0.769
**Nutritional Status**							
underweight	6	(2.2)	4	(66.7)	2	(33.3)	0.273
normal	110	(41.3)	65	(59.1)	45	(40.9)	
overweight	92	(33.5)	59	(64.1)	33	(35.9)	
obese	61	(23.0)	46	(75.4)	15	(24.6)	
**BMI (mean**,** SD)**	26.7	(4.8)	27.2	(4.9)	25.7	(4.4)	**0.018**
**Smoking status**							
current	71	(26.4)	44	(62.0)	27	(38.0)	0.836
previous	71	(26.4)	47	(66.2)	24	(33.8)	
never	126	(47.0)	82	(65.1)	44	(34.9)	
**Self-perceived general health**							
Very good or good	155	(57.6)	104	(67.0)	51	(33.0)	0.112
Fair	89	(33.0)	56	(63.0)	33	(37.0)	
Poor or very poor	24	(8.9)	13	(54.1)	11	(45.9)	
**Long standing health problem**							
Yes	89	(33.1)	64	(71.9)	25	(28.1)	0.245
**General activity limitation**							
present	68	(25.3)	44	(64.7)	24	(35.3)	0.249
**Well-being score (mean**,** SD)**	7.6	(1.6)	7.7	(1.5)	7.2	(1.5)	**0.007**

MVPA, moderate to vigorous physical activity. The values are presented as numbers and percentages, means (SDs) or medians (IQRs). Differences in the frequency distributions of variables by home gardening status were analyzed with Chi square tests or Fisher’s exact tests. Differences in continuous variable distribution by home gardening status were tested by t tests or the independent-samples Mann–Whitney U test.

### Associations between home gardening and meeting the recommended daily fruit and vegetable intakes

The associations between home gardening and meeting the recommended guidelines for fruit and vegetable intake are shown in Table 3. The measures of associations (Odds ratios) were adjusted for selected sociodemographic variables (age, sex, educational level, marital status, household composition, and settlement location) in a multivariate logistic regression model. Participants with a home garden had more than four times higher odds of meeting the recommendations for daily fruit and vegetable intake compared to those without a home garden (AOR = 4.49, 95% CI 1.95–10.18). Having a home garden was associated with 3.59 greater odds (AOR 3.59, 95% CI 1.79–7.19) of meeting the daily fruit intake recommendation and 3.69 greater odds (AOR 3.69, 95% CI 1.57–8.85) of meeting the daily vegetable intake recommendation compared with not having a home garden. The associations of fruit and vegetable intake with sociodemographic variables revealed that women were half as likely to meet the daily recommendation of fruit and vegetable intake as males. (AOR 0.50, 95% CI 0.26–0.96).

**Table 3:** Associations of home gardening and sociodemographic variables with meeting the recommendations for fruit and vegetable intake.

Variables	Fruit and vegetable intake			Fruit intake		Vegetable intake	
	AOR (95% CI)		*p*-value	AOR (95% CI)	*p*-value	AOR (95% CI)	*p*-value
**Home garden**							
Do not have	1.00			1.00		1.00	
Have	**4.49 (1.95**,** 10.18)**		**< 0.001**	**3.59 (1.79**,** 7.19)**	**< 0.001**	**3.69 (1.57**,** 8.85)**	**0.003**
**Age**	1.00 (0.97, 1.03)		0.792	0.99 (0.97, 1.01)	0.443	0.98 (0.96, 1.04)	0.446
**Sex**							
Male	1.00			1.00		1.00	
Female	**0.50 (0.26**,** 0.96)**		**0.041**	1.09 (0.56, 1.93)	0.796	0.54 (0.29, 1.18)	0.089
**Education level**							
High	1.00			1.00		1.00	
Low	0.67 (0.35, 1.21)		0.163	0.83 (0.48, 1.44)	0.514	0.63 (0.32, 1.23)	0.175
**Marital status**							
Married		1.00		1.00		1.00	
Single		0.42 (0.16, 1.11)	0.084	0.84 (0.34,1.89)	0.678	0.36 (0.20, 1.11)	0.074
Divorced or widowed		0.46 (0.13, 1.59)	0.224	1.03 (0.39, 2.70)	0.965	0.84 (0.24, 2.99)	0.789
Living with partner		0.46 (0.16, 1.27)	0.141	0.45 (0.18, 1.10)	0.093	0.39 (0.12, 1.23)	0.113
**Children under 18**							
No	1.00			1.00		1.00	
Yes	0.99 (0.64, 1.76)		0.994	1.02 (0.55, 1.88)	0.954	1.02 (0.49, 2.10)	0.952
**Settlement location**							
rural	1.00			1.00		1.00	
suburb	1.01 (0.56, 1.88)		0.921	0.91 (0.51, 1.61)	0.758	1.37 (0.69, 2.74)	0.468

Fruit and vegetable intake was dichotomized by meeting the recommended daily intake of 5 servings/day; fruit intake by meeting the recommended 2 servings/day; and vegetable intake by meeting the recommended 3 servings/day. Associations between variables were analyzed by multivariable logistic regression. Odds ratios were adjusted for the variables displayed in the table (i.e. age, sex, educational level, marital status, children under 18 and settlement location).Adjusted odds ratios (AOR, 95% CI) and p-values are reported.

## Discussion

The aim of the study was to assess whether home gardening is associated with fruit and vegetable consumption in two rural settlements in northeastern Hungary. To our knowledge, this is the first quantitative study of this kind conducted in Hungary. The main finding was that there is a positive association between home gardening and meeting the recommended daily intake of fruits and vegetables. However, we did not find significant differences among participants’ physical activity, nutritional status, smoking, and perceived general health by home gardening status.

Our findings are consistent with the available evidence from several studies that have observed higher fruit and vegetable consumption among home gardeners^16,30^. Systematic reviews reported that gardening was associated with higher fruit and vegetable consumption^31–36^. However, it is important to note that most of these studies have focused on community gardening and have primarily been conducted in the United States and other high-income countries. In contrast, most of the research on home gardening has taken place in low- and middle-income countries, where the primary objective has been to enhance food security and improve micronutrient availability^13,15,20,22,33,37–39^. Garden-based interventions showed promise for improving not only child nutrition but other indicators of their health^36^. Across 16 reviews, most evidence showed that fruit and vegetable intake improves nutritional status and mediators of intake (knowledge, willingness to taste)^36^. Recent research has been increasingly addressing the influence of urban gardening, both community gardens and domestic gardens. In a review focusing on the effects of urban gardening, the following results were reported: greater fruit and vegetable consumption, better access to healthy foods, greater valuing of cooking, harvest sharing with family and friends, enhanced importance of organic production, and valuing of adequate and healthy food^35^. While another review of 30 agricultural interventions found that increased food production did not necessarily improve nutrition or health within participating households. The interventions reviewed included home gardening, livestock, mixed garden and livestock, cash cropping, and irrigation. Nutrition was improved in 11 of 13 home gardening interventions, and in 11 of 17 other types of intervention. What is even more important is that they found that those projects which invested in human capital (especially nutrition education and consideration of gender issues) had a greater likelihood of effecting positive nutritional change, but such investment is neither sufficient nor always necessary to effect change^34^. Supporting and promoting community and home gardening may represent a potential approach to addressing food shortages that affect many parts of the world. It has been reported that fruit and vegetable consumption among households engaged in gardening doubled, reaching the daily intake recommended by US dietary guidelines^33^.

One study utilized a multi-level modeling approach to analyze trends and motivations behind personal food growth across Europe. Findings indicated that personal motivations, such as food security and environmental concerns, significantly influence individuals’ decisions to grow their own food^40^. Additionally, the research highlighted regional variations in these motivations, suggesting that local contexts play a crucial role in shaping food growing practices^40^.

Research on home gardening and fruit and vegetable intake has revealed several positive associations, such as an overall significant increase in fruit and vegetable consumption^11,30,37,41^, an increase in green leafy vegetable intake that can help reduce vitamin-A deficiency^38,39,42,43^, and a lower BMI^16^. Moreover, the benefits of home gardening may also benefit people other than the households that are enrolled. Families and neighbors may share what they produce, and second, admiring other people’s work and garden can lead to starting a garden by themselves^15^.

## Study limitations

The results of this study need to be considered in the context of its limitations. The use of data from cross-sectional studies limits the ability to determine whether home gardens are causally linked to fruit and vegetable consumption. Although the observed association was strong, the wide confidence interval reflects variability and limited precision, which is likely due to the small sample size. Further research involving larger sample sizes is needed to confirm these results. Furthermore, home gardening was assessed as a binary variable (yes/no), which does not capture heterogeneity in garden size, crop variety, productivity, seasonal availability, or the intensity of gardening activities. As a result, the observed association may mask important differences in the extent to which various forms of home gardening contribute to fruit and vegetable intake. As our study used a convenience sample of adults from rural and suburban settlements in Northeastern Hungary, the prevalence estimates apply specifically to this study population, and the results may not be applicable to other areas and are not nationally representative. The proportion of households sampled was higher in the rural than in the suburban area, which may result in biased results. Additionally, the data collected is self-reported, which could lead to measurement bias. For example, self-reported weight or height may not be accurate, and participants may over- or underreport their consumption of fruits and vegetables. The survey was only administered to one adult per household, which might not have allowed for capturing variations in dietary intake, nutritional knowledge or physical activity within households. The variation of diet due to seasonal changes also can influence the dietary intake of participants. Seasonality may also influence fruit and vegetable consumption. If the data collection had taken place during the summer–autumn season, when garden produce is at its most abundant, the observed associations would likely have been stronger, as gardeners would have consumed their usual amounts alongside their own produce. This potential measurement bias may have led to an underestimation of the true effect. Furthermore, there is a possibility of volunteer bias, and the study did not include adults without internet access. Although internet access is widespread in Hungary, even in rural areas, this does not eliminate the possibility of sampling bias. Despite these limitations, these results may suggest that home gardening has a positive effect on fruit and vegetable consumption.

## Conclusions

Our findings demonstrate a strong association between home gardening and meeting recommended daily fruit and vegetable intake in rural Northeast Hungary. By providing empirical evidence from a population with particularly low baseline consumption, this study extends existing literature and highlights home gardening as a potentially important, context-appropriate strategy for improving dietary quality in rural settings. The observed strength of the association suggests that promoting home gardening may represent a feasible public health approach to increasing fruit and vegetable intake. Future longitudinal and intervention studies are needed to establish causality and to assess the effectiveness of gardening-based interventions.

## Data Availability

The data that support the findings of this study are available from the corresponding author on reasonable request.

## Abbreviations

AOR: Adjusted odds ratio
BMI: Body mass index
CI: Confidence intervals
EHIS: European Health Interview Survey
EHIS-PAQ: European Health Interview Survey Physical Activity Questionnaire
FV: Fruits and vegetables
GBD: Global Burden of Disease
HG: Home gardening
IQR: Interquartile range
MVPA: Moderate to vigorous physical activity
OR: Odds ratio
PA: Physical activity
SD: Standard deviation
